# Post-infarction Left Ventricular Free Wall Rupture Diagnosed by Contrast-Enhanced Computed Tomography: A Case Report

**DOI:** 10.7759/cureus.52127

**Published:** 2024-01-11

**Authors:** Yuya Morimoto, Shinobu Tamura, Shuji Kawashima, Shigeaki Inoue

**Affiliations:** 1 Emergency and Critical Care Medicine, Wakayama Medical University, Wakayama, JPN

**Keywords:** contrast-enhanced computed tomography, echocardiography, left ventricular free wall rupture, impaired consciousness, st-segment elevation myocardial infarction

## Abstract

A 70-year-old Japanese woman with hypertension, dyslipidemia, and diabetes mellitus complained of abdominal discomfort and vomiting and was brought to our emergency department by ambulance two days later with impaired consciousness. Her vital signs suggested shock with a heart rate of 120 bpm. Electrocardiogram and initial transthoracic echocardiography suggested an inferior wall ST-elevation myocardial infarction, but the altered consciousness was inconsistent. Contrast-enhanced computed tomography was urgently performed to further clarify the cause. It revealed pericardial effusion and apparent extravasation from the left ventricular wall, confirming the early definitive diagnosis of left ventricular free wall rupture. The patient underwent successful emergent surgical repair without sequelae. Differential diagnosis of left ventricular free wall rupture is important in patients with ST-elevation myocardial infarction and impaired consciousness. Contrast-enhanced computed tomography allows early diagnosis and treatment of this life-threatening complication.

## Introduction

Mechanical complications after myocardial infarction (MI) include mitral regurgitation associated with papillary muscle rupture, ventricular septal defect, pseudoaneurysm, and free wall rupture [1]. Among them, rupture of the left ventricular free wall (LVFWR) is the most common and reportedly occurs in approximately 2% of patients with post-MI, although the true incidence rate is unknown [1,2]. LVFWR usually results in cardiac tamponade due to hematoma expansion into the pericardial space and is one of the most life-threatening complications after MI, with postoperative in-hospital mortality rates of 35% [1,2]. Early diagnosis and treatment of LVFWR in patients after infarction is therefore crucial yet challenging [2]. Transthoracic echocardiography (TTE) is considered to be the gold standard diagnostic tool and has a sensitivity of 100% and specificity of 93% for the diagnosis of LVFWR [3-6]. However, an accurate diagnosis of LVFWR is sometimes difficult due to the early stage of the disease or due to the examiner’s skill level. Herein, we describe the case of a patient with an undetermined level of consciousness in whom LVFWR after inferior MI was definitively diagnosed by contrast-enhanced computed tomography (CECT) in the emergency department.

## Case presentation

A 74-year-old Japanese woman presented with abdominal discomfort and vomiting. She had no chest pain or other radiating pain. The next morning, she had general discomfort and nausea but stayed at home. That night (day two of illness), she suddenly lost consciousness and was taken by ambulance to our emergency department. She had a medical history of rheumatoid arthritis, hypertension, dyslipidemia, and diabetes mellitus. She was regularly treated with oral loxoprofen (200 mg/day), prednisolone (5 mg/day), propranolol (10 mg/day), and rosuvastatin (10 mg/day). These underlying diseases were well-controlled.

On arrival, her level of consciousness was 6 on the Glasgow Coma Scale (E1V1M4). Her body temperature was 36.0°C, she had a heart rate of 120 beats per minute, blood pressure of 82/64 mmHg, and oxygen saturation of 94% on 9 L/min of oxygen via a non-rebreather mask. On physical examination, her pupils were bilaterally mydriatic and reactive to light without pupillary fixation. She had a mild cold sweat but no edema in the extremities. No extra heart sounds or murmurs were auscultated. Pulmonary examination revealed no rales or wheezing. Based on these findings, we clinically suspected that she had cardiogenic shock.

**Figure 1:**
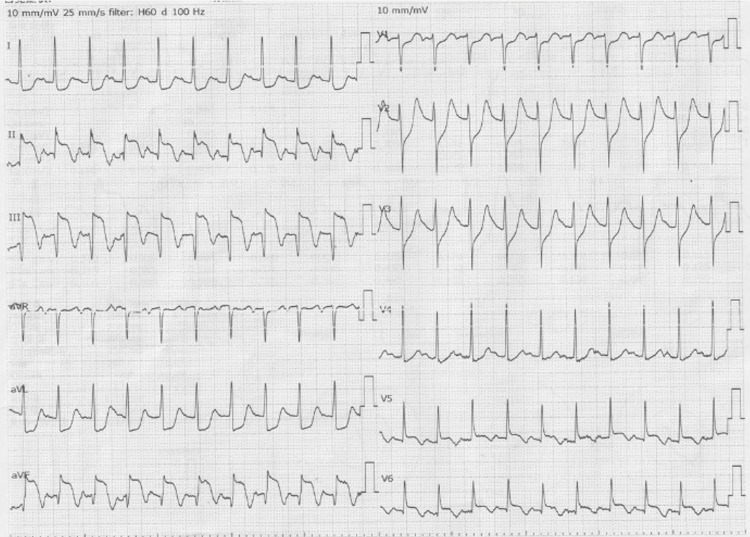
Electrocardiogram showed sinus tachycardia and ST-segment elevations in inferior leads, including II, III, aVF, and ST-segment depression in anterior leads V1-V4.

The electrocardiogram (ECG) was in sinus rhythm with ST segment elevation in leads II, III and aVF and ST segment depression in leads V1-4 (Figure 1). Bedside TTE by the emergency physician (a non-cardiologist) showed a left ventricular ejection fraction (LVEF) of 50% and abnormal inferior wall motion. In addition, the right ventricular wall motion was relatively preserved. We consulted with a cardiologist. Arterial blood gas analysis showed pH 7.410, carbon dioxide partial pressure 20.7 mmHg, oxygen partial pressure 146 mmHg, lactate 7.5 mg/dL, base excess -11.0 and bicarbonate 16.9 mmol/L. Laboratory tests revealed mild leukocytosis (white blood cell count 12.5 x 10^9^ /L) and elevated levels of aspartate aminotransferase (AST; 188 U/L), lactate dehydrogenase (LDH; 706 U/mL), creatinine (Cr; 1.92 mg/dl), creatine kinase (CK; 1,478 U/L) and C-reactive protein (CRP; 2.62 mg/dL) (Table 1). CK-myocardial band (CK-MB) and troponin I, specific myocardial markers, were also elevated (105 U/ml and 66 499.1 pg/ml, respectively) (Table 1). An inferior wall ST-elevation MI (STEMI) was therefore diagnosed within 30 minutes of arrival at the emergency department.

**Table 1:** Laboratory findings at the visit to our hospital

	Value	Reference range
Complete Blood Count		
White blood cells	12.5 x10^9^/L	3.3-8.6 x10^9^/L
Neutrophils	71.5 %	
Lymphocytes	24.7 %	
Red blood cells	4,230 x10^9^/L	3,860-4,920 x10^9^/L
Hemoglobin	13.1 g/dL	11.6–14.8 g/dL
Hematocrit	39.4 %	35.1-44.4 %
Mean corpuscular volume	93.1 fL	83.6-98.2 fL
Platelets	220 x10^9^/L	158-348 x10^9^/L
Coagulation System		
Activated partial thromboplastin time	24.3 sec	24-40 sec
Prothrombin time	11.7 sec	11-13 sec
Fibrinogen	453 mg/dL	160-350 mg/dL
D-dimer	4.70μg/ml	0-1μg/ml
Chemistry		
Aspartate aminotransferase	188 U/L	13-30 U/L
Alanine aminotransferase	30 U/L	7-23 U/L
Lactate dehydrogenase	706 U/L	124-222 U/L
Alkaline phosphatase	73 U/L	38-113 U/L
γ-Glutamyl Transpeptidase	20 U/L	9-32 U/L
Amylase	294 U/L	44-132 U/L
Total protein	6.1 g/dL	6.6-8.1 g/dL
Albumin	3.5 g/dL	4.1-5.1 g/dL
Total bilirubin	1.5 mg/dL	0.4-1.5 mg/dL
Creatinine	1.92 mg/dL	0.46-0.79 mg/dL
Blood urea nitrogen	22.1 mg/dL	8-20 mg/dL
Creatine kinase	1,478 U/L	41-153 U/L
Creatine kinase MB	105 U/L	0-12 U/L
Sodium	131 mmol/L	138-145 mmol/L
Potassium	4.3 mmol/L	3.6-4.8 mmol/L
Chloride	101 mmol/L	101-108 mmol/L
C-reactive protein	2.62 mg/dL	0.00-0.14 mg/dL
Troponin I	66,499.1 pg/ml	0-26.2 pg/ml

**Figure 2:**
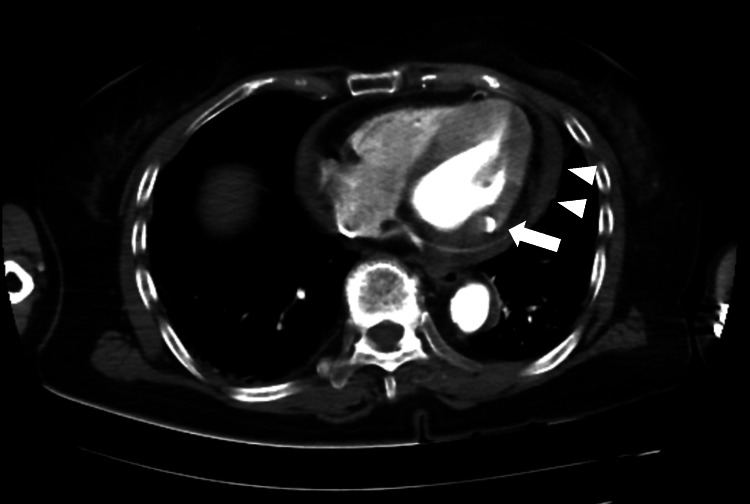
Contrast-enhanced computed tomography (CECT) imaging shows effusion from the left ventricle into the pericardial space (arrow) and pericardial effusion (arrowheads).

**Figure 3:**
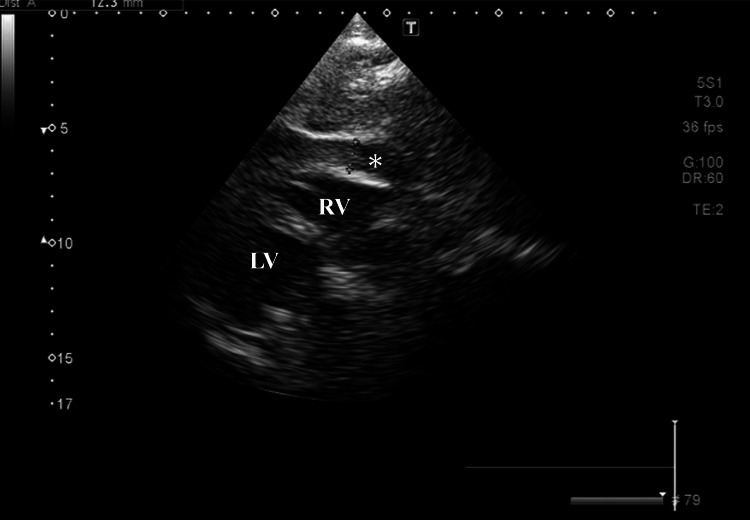
Transthoracic echocardiogram after the contrast-enhanced computed tomography (CECT) shows 13-mm pericardial effusion (asterisk). LV; left ventricle, RV; right ventricle.

Meanwhile, her altered consciousness was prolonged for more than one hour. At this point, additional differential diagnoses were considered, including intracranial lesions or aortic dissection. Thus, a CECT was performed immediately, despite the patient’s history of renal impairment. The CECT also showed abnormalities with inferior myocardial thinning, effusion from the left ventricle into the pericardial space (Figure 2 arrow), and pericardial effusion (Figure 2 arrowheads). These findings were consistent with a diagnosis of LVFWR probably complicated by STEMI. No aortic dissection, macrovascular, or intracranial lesions were found. Subsequently, TTE revealed a 13 mm pericardial effusion (Figure 3).

No apparent increase in pericardial effusion was observed on TTE in the emergency department. The patient was intubated and referred to the Department of Cardiovascular Surgery for emergent surgery after arterial line placement and intra-arterial balloon pumping (IABP). Within two hours of arrival, surgical evacuation of the intrapericardial hematoma and left ventricular repair for LVFWR was initiated. After reinforcement with TachoSil® patches and fibrin glue, the operation was completed. The patient was then admitted to the intensive care unit and weaned off the ventilator and IABP on postoperative day five. She had the complication of stage 4 chronic kidney disease. Angiographic dye has been known to cause worsening renal function in patients with underlying kidney disease. Thus, no additional coronary angiography was performed. She was transferred to a rehabilitation hospital on day 57 and discharged on day 122. At the time of writing, she has been doing well for more than a year.

## Discussion

LVFWR usually occurs within one week of MI, with the majority of cases occurring within the first 24 hours [7]. LVFWR can be classically classified into two anatomic-pathological types: oozing and blowout ruptures. A meta-analysis showed a lower operative risk in oozing-type LVFWR compared with blowout-type LVFWR (risk ratios: 0.47; 95% confidence interval: 0.33-0.67; p < .0001) [8]. A previous retrospective report from a single-center study described that the in-hospital survival rate for oozing-type LVFWR was higher than for blowout-type LVFWR (63.4% vs. 36.4%, respectively) [9]. Furthermore, a multi-center cohort study recently reported that oozing-type rupture was associated with a higher survival rate compared with blowout-type (65.2% vs 34.8%, respectively) [10]. In our patient’s case, an ischemic event probably occurred within 24 hours before the transfer. The initial bedside TTE showed poor motion of the inferior wall but no obvious pericardial effusion. Even after the definitive diagnosis of LFVWR, there was no observation of rapidly increasing effusion and circulatory collapse. The LFVWR in the present case is thus suggested to be an oozing-type rupture and it occurred in the early stage, allowing for a relatively favorable outcome.

Risk factors for the development of LVFWR after MI include age older than 65 years, female sex, arterial hypertension, no history of previous MI, anterior wall MI, transmural infarction, and ST-segment elevation or Q-wave development on initial ECG [11-15]. Meanwhile, metabolic comorbidities including diabetes mellitus, hypertension, and dyslipidemia, were not risk factors for the development of LVFWR [11-15]. Among these factors, our patient presented with advanced age, female sex, no history of previous MI, and ST-segment elevation; this indicated an increased risk of developing LVFWR. Early recognition of such risk factors in cases of STEMI, as in our case, may allow timely definitive diagnosis and surgical intervention for LVFWR. 

In a patient with STEMI, TTE is the fastest and most useful test for the prompt diagnosis of LVFWR, demonstrating pericardial effusion, reduced myocardial wall thickness, pericardial or epicardial thrombus, and the presence of cardiac tamponade [3-6]. Pericardial effusion is the most common finding, with a sensitivity of up to 100%, but has a specificity of 93% [3,5]. In clinical practice, it is difficult to differentiate LVFWR from pericarditis, bleeding from post-infarcted myocardium, and coronary perforation. On the contrary, the absence of pericardial effusion does not exclude the possibility of LVFWR. Accordingly, even in cases of STEMI without obvious pericardial effusion on initial TTE, the development of LVFWR should be considered if there are inexplicable symptoms.

In our case, in which the patient had shock and altered consciousness, we also suspected aortic dissection and subarachnoid hemorrhage with ST-segment changes. Despite the presence of renal dysfunction, CECT was performed to clarify these conditions, and left ventricular rupture and thin pericardial effusion were first observed. CECT is one of the most effective modalities to detect LVFWR and it is useful to exclude hemopericardium complicated by aortic dissection [16-18]. Indeed, case reports of post-MI LVFWR diagnosed by CECT, but not TTE, have recently increased [16-18]. Recently, a large retrospective cohort study found that the use of intravenous contrast was not associated with renal outcomes (either persistent acute kidney injury (AKI) at discharge or initiation of dialysis within 180 days) in patients with pre-existing AKI [19]. Even in the presence of renal impairment, CECT should be promptly recommended in suspected patients to avoid missing life-threatening conditions such as cardiac rupture complicating MI, aortic dissection, and subarachnoid hemorrhage. Our patient with post-MI LVFWR has survived and achieved normal condition despite the complication of stage 4 chronic kidney disease.

The initial management of post-MI LVFWR is to stabilize hemodynamics with pericardiocentesis and IABP [2]. As conservative strategies do not improve the prognosis, emergency surgical repair is usually the definitive treatment for LVFWR [2]. In our case, we suggest that IABP support helped to prepare the opportunity for surgical treatment. Prompt cardiac catheterization before surgery remains controversial, so there is the possibility of delayed treatment and surgery for LVFWR [20]. Surgical techniques for LVFWR include pericardial patch placement with either biological glue or epicardial sutures [2]. In our case, in which we performed sutureless repair, there were no complications such as re-rupture, left ventricular aneurysm, or pseudoaneurysm formation. 

## Conclusions

LVFWR is a rare but potentially fatal mechanical complication of post-MI and should be carefully considered in any patient with MI presenting with cardiogenic shock and multiple risk factors. TTE is considered to be the gold standard for diagnosis, but urgent CECT is also a useful modality to avoid misdiagnosis of this life-threatening complication.
